# Bilateral anophthalmia and intrahepatic biliary atresia, two unusual components of Fraser syndrome: a case report

**DOI:** 10.1186/s12884-020-03048-x

**Published:** 2020-06-10

**Authors:** Muhamad Zakaria Brimo Alsaman, Sarab Agha, Hala Sallah, Rayan Badawi, Mohammad Nour Kitaz, Abdullah Assani, Hamdi Nawfal

**Affiliations:** 1 Faculty of Medicine, University of Aleppo, Aleppo, Syria; 2 Department of Pathology, Faculty of Medicine, University of Aleppo, Aleppo, Syria; 3 Department of Embryology, Faculty of Medicine, University of Aleppo, Aleppo, Syria

**Keywords:** Fraser syndrome, Cryptophthalmos syndrome, Anophthalmia, Intrahepatic biliary atresia

## Abstract

**Background:**

Fraser syndrome or “cryptophthalmos syndrome” is a rare autosomal recessive disease. It is characterized by a group of congenital malformations such as: crytophthalmos, syndactyly, abnormal genitalia, and malformations of the nose, ears, and larynx. Although cryptophthalmos is considered as a main feature of Fraser syndrome, its absence does not exclude the diagnosis. Clinical diagnosis can be made by Thomas Criteria. Here we present the first documented case of Fraser Syndrome in Aleppo, Syria that is characterized by bilateral anophthalmia and intrahepatic biliary atresia.

**Case presentation:**

During pregnancy, several ultrasound scans revealed hyperechoic lungs, ascites, and unremarkable right kidney at the 19th-week visit; bilateral syndactyly on both hands and feet at the 32nd-week visit. On the 39th week of gestation, the stillborn was delivered by cesarean section due to cephalopelvic disproportion. Gross examination showed bilateral anophthalmia, bilateral syndactyly on hands and feet, low set ears, and ambiguous genitalia. Microscopic examination of the lung, spleen, liver, ovary, and kidneys revealed abnormalities in these organs.

**Conclusion:**

The diagnosis of Fraser syndrome can be made prenatally and postnatally; prenatally by ultrasound at 18 weeks of gestation and postnatally by clinical examination using Thomas criteria. Moreover, intrahepatic biliary atresia was not described previously with Fraser syndrome; this recommends a more detailed pathologic study for Fraser syndrome cases.

## Background

Fraser syndrome or cryptophthalmos syndrome [1], also known as Fraser-Francois syndrome, Meyer-Schwickerath’s syndrome, Ulrich-Feichtiger syndrome or cryptophthalmos-syndactyly syndrome [2, 3], is a rare autosomal recessive disease characterized by a group of congenital malformations, such as cryptophthalmos; syndactyly; abnormal genitalia; malformation of the nose, ears, and larynx; cleft lip; skeletal defects; umbilical hernia; mental retardation, and renal agenesis [1].

There are extremely few reported cases of Fraser syndrome with anophthalmia [4–7]; as Cryptophthalmos accounts for 88% of ocular malformations [8].

Genetically, mutations in FRAS1, FREM1, FREM2, and GRIP1 are responsible for the disease. These genes are essential for the regulation of epidermal-basement membrane adhesion and organogenesis during the embryonic period [9–11].

Fraser syndrome was first described by George Fraser in 1962 [6]. In which 25% of affected children are stillborns [12]. The estimated prevalence of Fraser syndrome is below 0.43 per 100,000 live-born infants and 11.06 per 100,000 stillbirths [13].

The diagnosis can be made prenatally by ultrasound, or clinically using Thomas criteria- two major criteria and one minor criterion, or one major criterion and four minor criteria are sufficient for diagnosis [1].. The major criteria are: cryptophthalmos, syndactyly, abnormal genitalia, and a sib with cryptophthalmos syndrome. The minor criteria are: cleft lip and/or palate, skeletal defects, and congenital malformation of the nose, ears, and larynx [1].

Prognosis of Fraser Syndrome depends on type and severity of the malformations and their possibility to be fixed [3, 14]..

Here we present the first documented case of Fraser syndrome in Aleppo, Syria that is characterized by bilateral anophthalmia and intrahepatic biliary atresia.

## Case presentation

A 19-year-old woman (G2P1), married as a non-consanguineous marriage, with no past medical or surgical history, came to the outpatient prenatal clinic for routine visits.

**Fig. 1:**
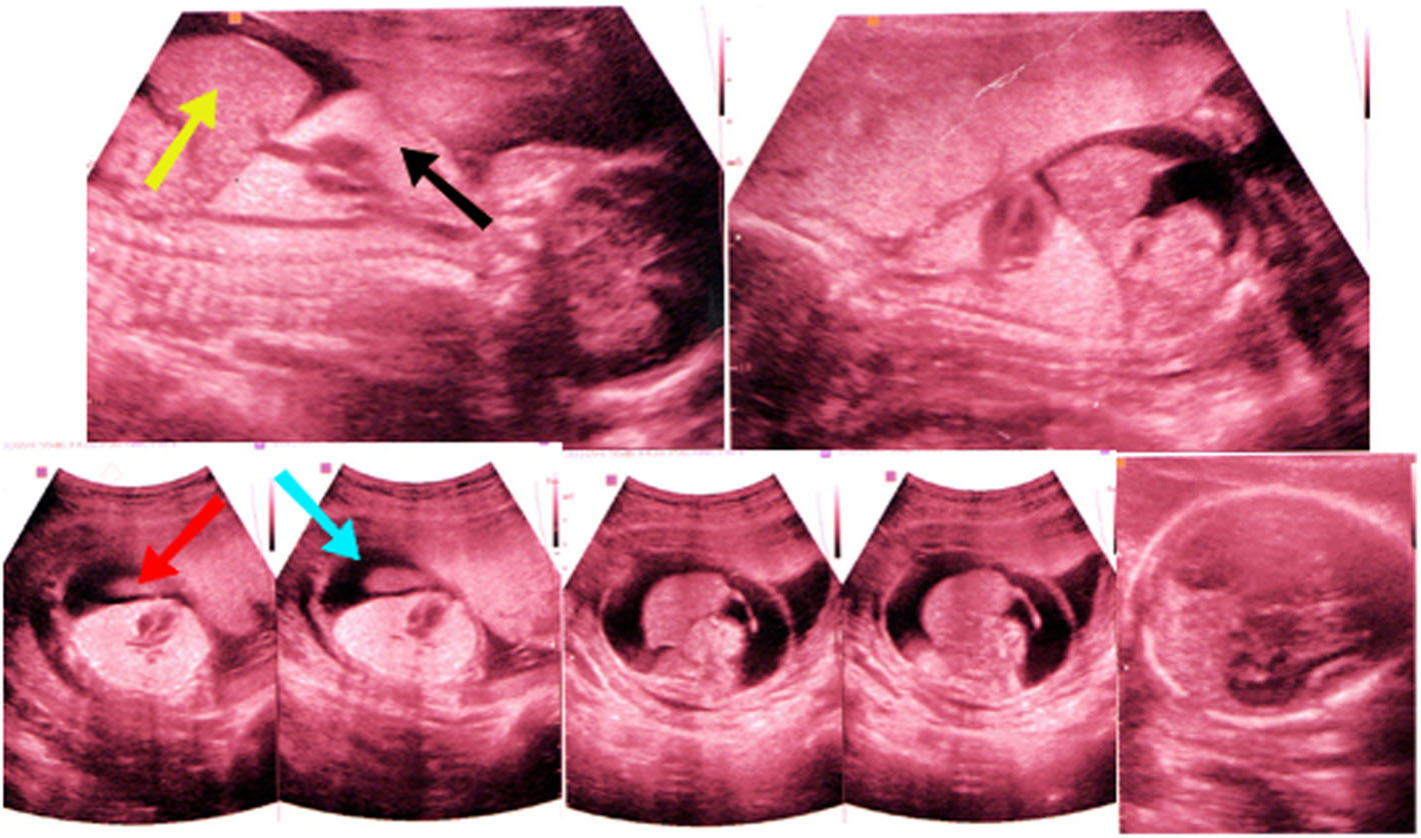
Ultrasound scans of the 20th-week visit: yellow arrow indicates liver, black arrow indicates lung, red arrow indicates kidney and blue arrow indicates ascites

**Fig. 2:**
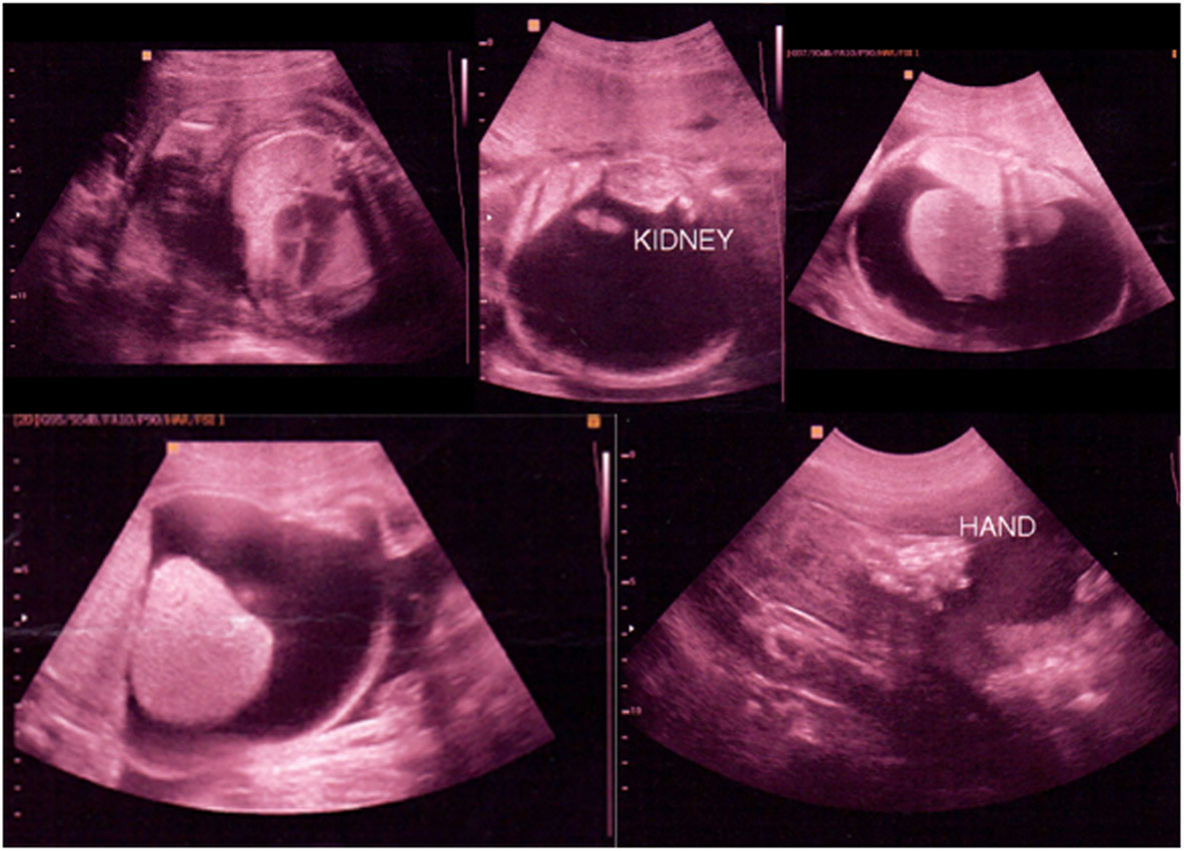
Ultrasound scans of the 25th-week visit showed: relative shortness in upper and lower limbs, nuchal edema, bilateral syndactyly on both hands and feet, hydrops fetalis, enlargement of the lungs, and cardiac compression

Prenatal ultrasound scans of several visits revealed:

- Hyperechoic lungs, ascites, unremarkable kidney (right), normal sucking reflux and a three-vessels umbilical cord in the 20th-week visit (Fig. 1).
- Relative shortness in upper and lower limbs, nuchal edema, bilateral syndactyly on both hands and feet, hydrops fetalis, the abdominal circumference was 420 mm, enlargement of the lungs, and cardiac compression. The ultrasound estimated weight was 4000 g in the 25th-week visit (Fig. 2).

**Fig. 3:**
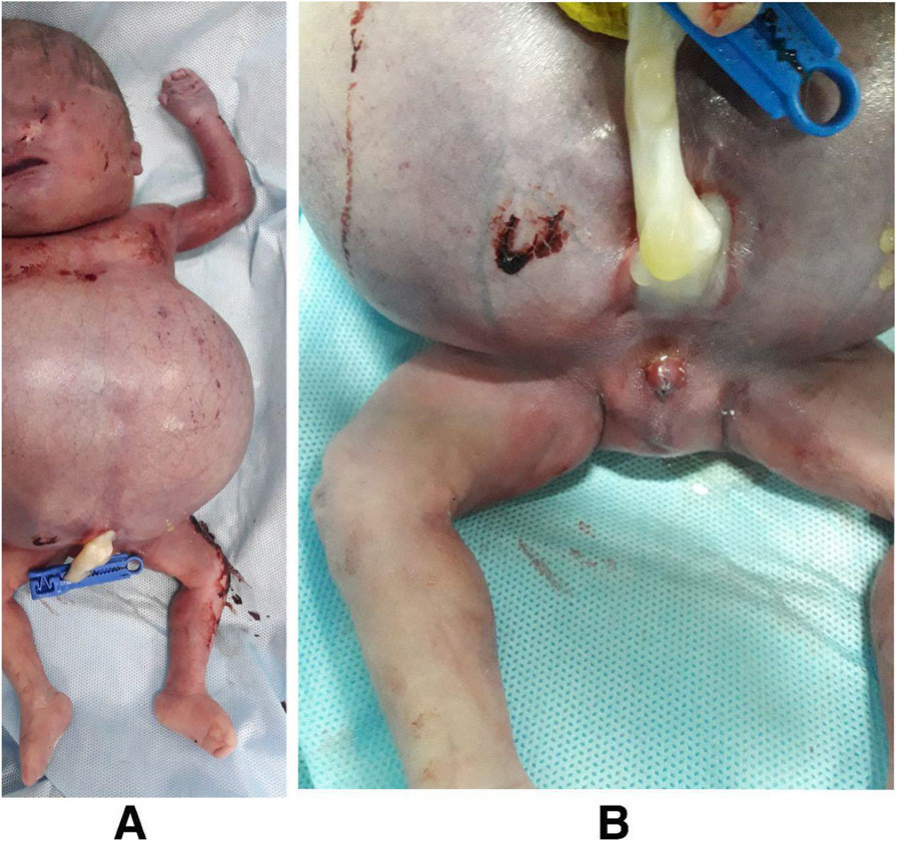
Stillborn with bilateral anophthalmia, pseudo-hypertelorism, low-set ears, flat nasal bridge, bilateral syndactyly on hands and feet, cutaneous and subcutaneous edema, large-volume ascites (A) and ambiguous genitalia (B)

After 39 weeks of gestation, the mother underwent a cesarean section for cephalopelvic disproportion. The fetus was stillborn due to respiratory insufficiency, weighing 3600 g. Gross examination showed multiple abnormalities including:

- Bilateral anophthalmia, pseudo-hypertelorism, low-set ears, flat nasal bridge, bilateral syndactyly on hands and feet, cutaneous and subcutaneous edema, large-volume ascites, (Fig. 3 a) and ambiguous genitalia (Fig. 3 b).

**Fig. 4:**
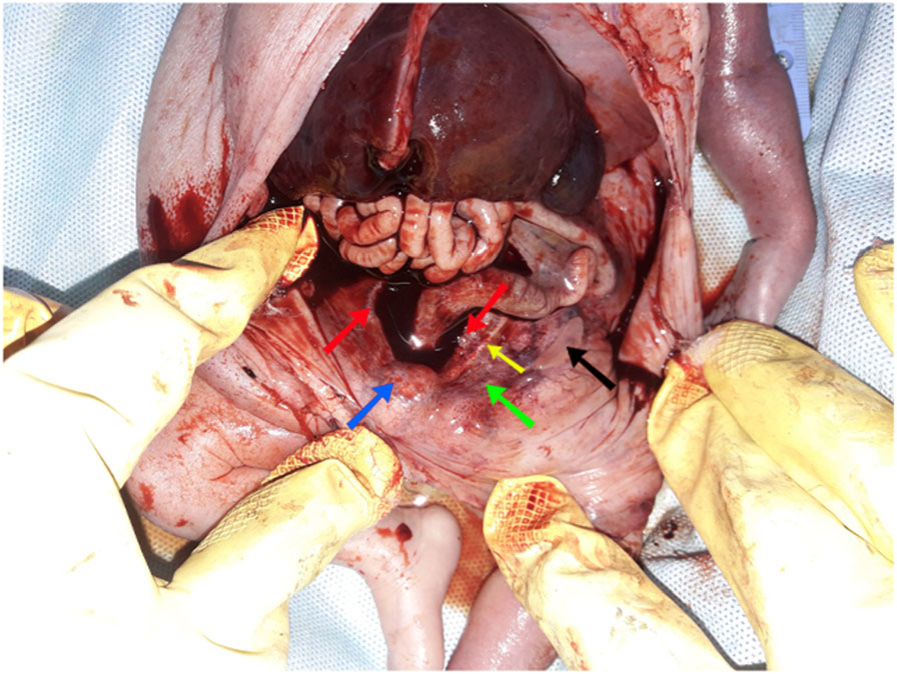
Red arrows indicate right and left common iliac arteries, blue arrow indicates bladder, yellow arrow indicates the left ureter, green arrow indicates Mullerian duct and black arrow indicates gonad. The figure shows an absent right ureter and kidney

Autopsy revealed: normal airways, lung enlargement, no significant cardiac abnormalities, unilateral renal agenesis (right), a gonad and Mullerian structure were found on the left posterior pelvic wall, and the most outstanding find was the congested liver capsule which led us to do pathophysiologic study to find out the reason (Fig. 4).

**Fig. 5:**
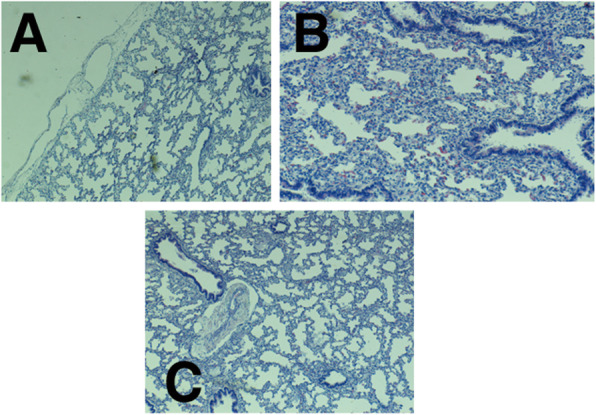
Dilated pleural lymphatic vessels (A), interstitial fibroblast hypertrophy (B) and vascular wall thickening in the lung (C)

**Fig. 6:**
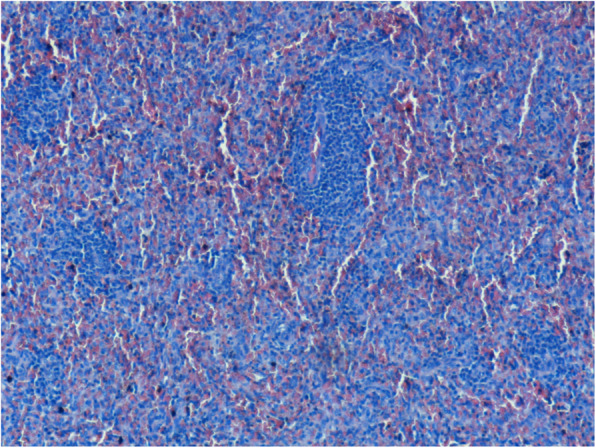
Congestion of red pulp with hemosiderin accumulation and immature white pulp of the spleen

Microscopic examination of multiple sections of the lung, spleen, liver, ovary, and kidneys showed:

- Dilated pleural lymphatic vessels (Fig. 5 a), interstitial fibroblast hypertrophy (Fig. 5 b) and vascular wall thickening in the lung (Fig. 5 c).
- Congestion of red pulp with hemosiderin accumulation and immature white pulp of the spleen (Fig. 6).

**Fig. 7:**
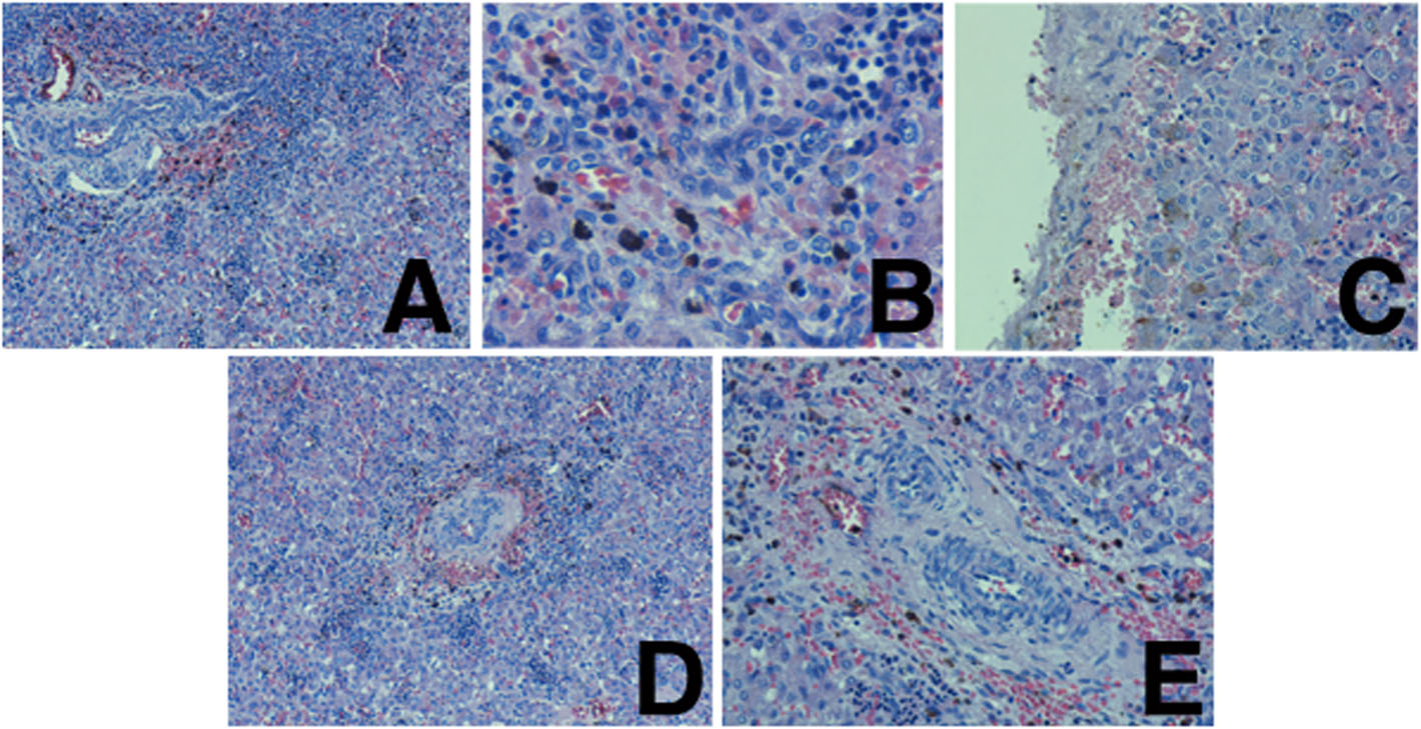
Cholestasis with biliary atresia (A, D, C) and biliary plugs (B, C), portal spaces fibrosis (A, D, E) with diffused congestion and diffused chronic inflammatory infiltrations with lymphocytes in the liver (B)

Cholestasis with biliary atresia (Fig. 7 a, d, c) and biliary plugs (Fig. 7 B, C), portal spaces fibrosis (Fig. 7 a, d, e) with diffused congestion and diffused chronic inflammatory infiltrations with lymphocytes in the liver (Fig. 7 B).

**Fig. 8:**
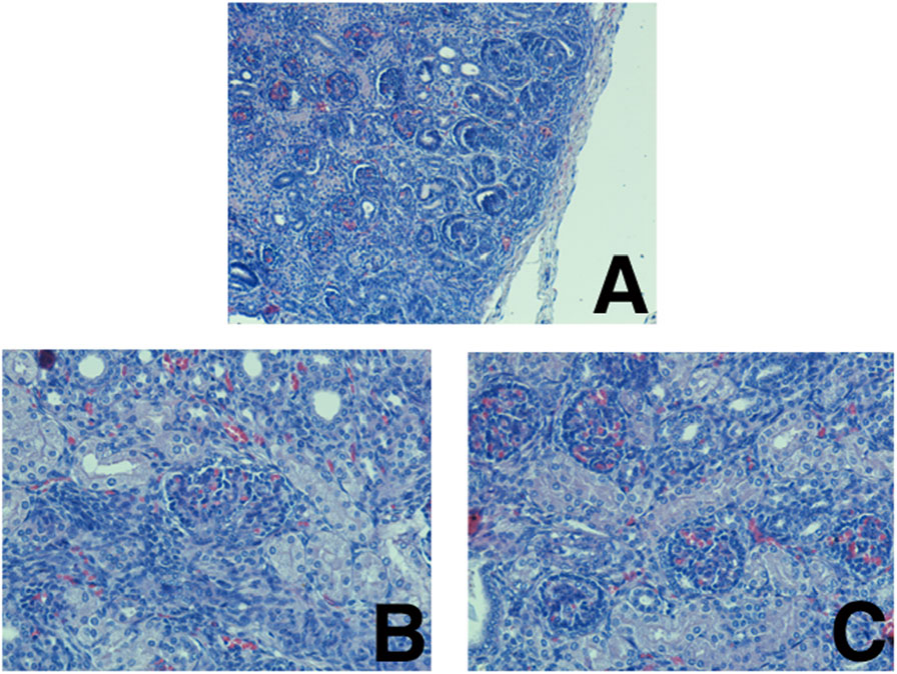
Subcapsular nephrogenic structures (A) and glomerular changes, such as decreasing subcapsular spaces (B) and changing in the simple squamous epithelium of the outer parietal layer of the glomerular capsule into cuboidal epithelium in the kidney (C)

While kidney sections revealed increased subcapsular nephrogenic structures (Fig. 8 a) and glomerular changes, such as decreasing subcapsular spaces (Fig. 8 b) and changing in the simple squamous epithelium of the outer parietal layer of the glomerular capsule into cuboidal epithelium (Fig. 8 c).

**Fig. 9:**
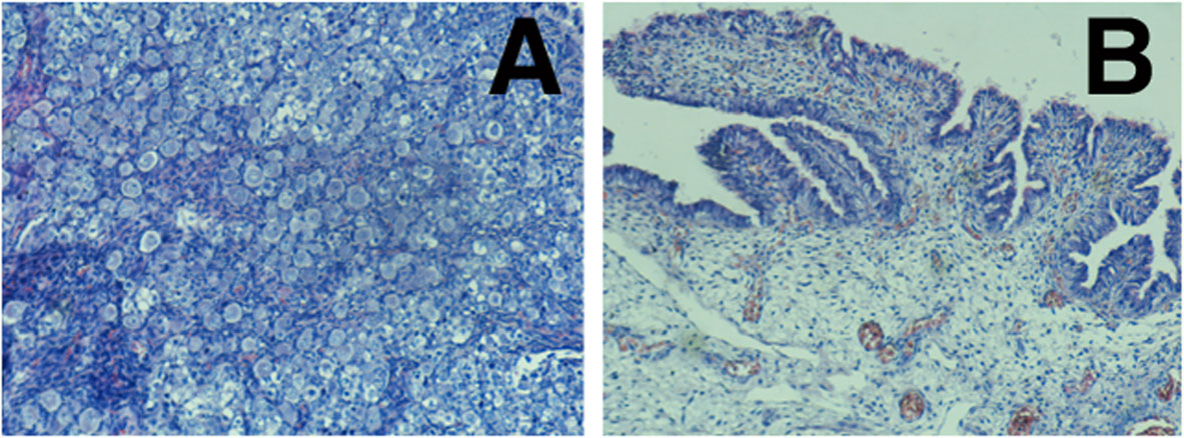
Clusters of crowded immature primordial follicles (A) and the Mullerian duct derivative was made up of epithelium resembling that of the Fallopian tube (B)

As for the gonads, there were clusters of crowded immature primordial follicles (Fig. 9 a) and the Mullerian duct derivative was made up of epithelium resembling that of the Fallopian tube (Fig. 9 b).

Subsequently, based on microscopic examination of the lung and the presence of congenital malformations such as anophthalmia, bilateral syndactyly and renal agenesis, we suspected: plexiform pulmonary arteriopathy, Fraser Syndrome and Lenz microphthalmia syndrome as differential diagnosis for our case, but Fraser Syndrome was the most suitable one depending on Thomas et al. criteria.

The parents refused the genetic tests for the stillborn due to familial reasons.

## Discussion and conclusion

Fraser Syndrome (OMIM 219000) is a rare autosomal recessive disease; probable mutations responsible for this disease are: FRAS1, FREM1, FREM2, and GRIP1 [9–11].

There are more than 250 reported cases of Fraser syndrome in medical literature [15].

Thomas et al. created the diagnostic criteria for Fraser syndrome [1]. The major criteria are: crytophthalmos, syndactyly, abnormal genitalia, and a sib with cryptophthalmos syndrome. The minor criteria are: cleft lip and/or palate, skeletal defects, and congenital malformation of the nose, ears, and larynx. Two major criteria and one minor criterion, or one major criterion and four minor criteria are sufficient for diagnosis [1].. alternatively, According to Slavotinek A et al. the diagnosis of Fraser syndrome can be made by the presence of one major criterion and one minor criterion [8].

In our case, the stillborn neonate had: bilateral anophthalmia, bilateral syndactyly (major) on hands and feet, ambiguous genitalia (major), low set ears (minor), unilateral renal agenesis (minor), and malformation of the nose (minor).

We have two major and three minor criteria that clinically affirm Fraser syndrome diagnosis according to Thomas and Slavotinek et al. criteria.

Cryptophthalmos; a continuous skin covering a normal or malformed eye [16], is the most common feature of Fraser Syndrome; it accounts for 88% of ocular malformations. However, other malformations can be present instead, such as microphthalmia; a small eye with normal eyelids [17], corneal opacification, and anophthalmia [8].

Anophthalmia; Inexistence of the eye [16], accounts for 6% of ocular malformations, and there are extremely few reported cases of Fraser syndrome with anophthalmia [4–6, 8].

Plexiform pulmonary arteriopathy and lenz microphthalmia syndrome were also differential diagnoses for our case, but the presence of congenital malformations led us to exclude plexiform pulmonary arteriopathy.

Lenz microphthalmia syndrome is characterized by anophthalmia or microphthalmia, in addition to mental retardation; external ear; digital, cardiac, skeletal and urogenital anomalies. It differs from Fraser syndrome by the presence of spinal and dental anomalies [18].

The definitive diagnosis of Fraser syndrome is only made by genetic analysis, detecting for FRAS1, FREM1, FREM2, and GRIP1 genes mutations, and this was a limitation in our case because the parents refused the genetic tests for the stillborn.

Prenatal diagnosis of Fraser syndrome is possible by ultrasound at 18 weeks of gestation with the following diagnostic signs:Hyperechogenic lungs, laryngeal stenosis/atresia, oligohydramnios, ascites, renal agenesis/dysplasia, microphthalmia/hypertelorism, hydrocephalus, syndactyly, ear defects and ambiguous genitals [19–21].

Prenatal diagnosis of Fraser syndrome provides a better management for infants with severe malformations in the respiratory tract [21].

In our case, prenatal ultrasound scans revealed: hyperechoic lungs, ascites, and an unremarkable right kidney at the 19th-week visit and bilateral syndactyly on both hands and feet at the 32nd-week visit.

Nevertheless, prenatal diagnosis is limited in relation to incidence of prenatal diagnosis obtained of Fraser syndrome. Berg et al. reported that prenatal diagnosis of FS was only in 43.75% (range 16–23 weeks of gestational age; sample size: 8) [19].

However, the rarity and the lack of knowledge of this syndrome in Syria made the diagnosis challenging. So that, the final probable diagnosis was made after birth.

Neonatal Cholestasis can occur due to deficiency in the excretion of bile, either for obstructive reasons or due to lack of bile secretion into the bile canaliculus [22].

Intrahepatic biliary atresia (paucity of intrahepatic bile ducts) can cause neonatal cholestasis [23]. It occurs in syndromic or non-syndromic form. The syndromic form, such as Alagille syndrome [24]. The non-syndromic form is associated with chromosomal disorders, metabolic or viral diseases, altered bile acid metabolism, and cystic fibrosis [25].

To sum up, intrahepatic biliary atresia was not described previously with Fraser Syndrome; this recommends a more detailed pathologic study for Fraser syndrome cases.

## Data Availability

All data generated or analysed during this study are included in this published article and its supplementary information files.
